# Arterial Calcification Disappearance in Breast Imaging: A Key Indicator for Transition to Invasive Ductal Carcinoma

**DOI:** 10.3390/diagnostics14070727

**Published:** 2024-03-29

**Authors:** Arisa Sato, Tomoyuki Fujioka, Iichiroh Onishi, Emi Yamaga, Leona Katsuta, Kazunori Kubota, Yuichi Kumaki, Toshiyuki Ishiba, Goshi Oda, Ukihide Tateishi

**Affiliations:** 1 Department of Diagnostic Radiology, Tokyo Medical and Dental University, 1-5-45, Yushima, Bunkyo-ku, Tokyo 113-8519, Japan; 2 Department of Radiology, Nitobe Memorial Nakano General Hospital, 4-59-16, Chuo, Nakano-ku, Tokyo 164-8609, Japan; 3 Department of Comprehensive Pathology, Tokyo Medical and Dental University, 1-5-45 Yushima, Bunkyo-ku, Tokyo 113-8510, Japan; 4 Department of Radiology, Dokkyo Medical University Saitama Medical Center, 2-1-50, Minamikoshigaya, Koshigaya 343-8555, Saitama, Japan; 5 Department of Surgery, Breast Surgery, Tokyo Medical and Dental University, 1-5-45, Yushima, Bunkyo-ku, Tokyo 113-8510, Japan

**Keywords:** breast cancer, mammography, ultrasound, arterial calcification, disappearance

## Abstract

A woman in her 70s, initially suspected of having fibroadenoma due to a well-defined mass in her breast, underwent regular mammography and ultrasound screenings. Over several years, no appreciable alterations in the mass were observed, maintaining the fibroadenoma diagnosis. However, in the fourth year, an ultrasound indicated slight enlargement and peripheral irregularities in the mass, even though the mammography images at that time showed no alterations. Interestingly, mammography images over time showed the gradual disappearance of previously observed arterial calcification around the mass. Pathological examination eventually identified the mass as invasive ductal carcinoma. Although the patient had breast tissue arterial calcification typical of atherosclerosis, none was present around the tumor-associated arteries. This case highlights the importance of monitoring arterial calcification changes in mammography, suggesting that they are crucial indicators in breast cancer diagnosis, beyond observing size and shape alterations.

Breast cancer is one of the most common forms cancers in women, and early detection plays a crucial role in its treatment and prognosis. Common screening methods include ultrasound and mammography, which are widely used to detect abnormalities in breast tissue [1]. Despite the effectiveness of these methods, some cases progress in unusual ways. In our study, we report a rare instance observed during follow-up, in which arterial calcification, initially identified in mammography, disappeared. This report not only shares this unique case, but also explores the potential mechanisms underlying this phenomenon through an extensive literature review. A key aspect of our study includes a detailed comparison between breast imaging and pathological findings, shedding light on the correlations and discrepancies between these diagnostic modalities.

The patient was a 70-year-old woman who had previously been treated for hepatitis C, but was not undergoing any treatment at the time, including medication. She had no other significant medical history, and there was no family history of breast or ovarian cancer. She underwent regular screenings, including ultrasound and mammography. Initial examinations revealed a well-defined, oval-shaped mass with clear borders in her breast, which raised suspicion of fibroadenoma (Figure 1 and Figure 2). Over several years, annual ultrasound and mammography screenings showed no appreciable alterations in the size or shape of the mass, sustaining the fibroadenoma diagnosis.

During the fourth-year follow-up, although there was no apparent change in its size, a subsequent ultrasound examination revealed slight enlargement and irregularities at the periphery of the mass (Figure 3). Surprisingly, a review of previous mammography images revealed the disappearance of pre-existing arterial calcifications within and around the mass. Furthermore, upon examining the mammography images over time, a gradual disappearance of calcifications was observed (Figure 4).

Pathological examination results confirmed that the breast mass was an invasive ductal carcinoma (Figure 5). Surgical pathology also indicated thick blood vessels within the tumor, suggesting a correlation with the initially observed calcified artery. However, there were no clear signs of tumor invasion into the blood vessels. Notably, although the patient had calcifications (atherosclerosis) in the arteries of her normal breast tissue, calcifications were not observed within or around the tumor-associated arteries (Figure 6).

This case, although initially diagnosed as fibroadenoma and observed over time, eventually presented with invasive ductal carcinoma. Although the alteration in the mass’s size over time was limited, the disappearance of arterial calcification within and around the tumor was observed. Although rare, there have been past reports of cases where calcification within the ducts disappeared with the emergence of invasive cancer [2,3], so in this case, the disappearance of calcification could be related to the progression of breast cancer, and the potential mechanisms of this were examined. We considered the mechanism of tumor invasion into the arterial wall as a cause of calcification disappearance. However, pathological evidence to support this hypothesis was not found.

As the malignancy of a tumor increases, blood perfusion within the tumor becomes heterogeneous. This is due to the presence of regions with high blood perfusion, moderate perfusion, low perfusion, and zero perfusion or necrotic tumor zones [4]. Such changes in blood flow are attributed to alterations in vascular structure caused by rapid tumor growth. Normally, arterial wall calcification occurs in a stable blood flow condition. However, as breast cancer progresses, an increase in blood perfusion heterogeneity may lead to the dissolution of existing calcifications. Additionally, the tumor may secrete cytokines that promote angiogenesis, which could also influence the disappearance of calcification [5].

Although not breast cancer, a previous case reported calcification loss in patients with glioma, owing to local malignant alterations. The authors hypothesized that, in the presence of malignant tumors, calcifications may disappear due to a decrease in microenvironmental pH [6]. This factor could have been involved in the arterial wall calcification observed in our patient. It is important to note, however, that our analysis of the mechanisms behind this phenomenon is purely speculative and based on a literature review. Proving such mechanisms conclusively is challenging, and there are inherent limitations in confirming these hypotheses without further detailed research and case accumulation.

Calcification within the breast is a common finding, and mammography is the most effective method for its detection. Additionally, although calcification within the mammary ducts can occur in benign and malignant tumors, their nature can often be inferred from their distribution and morphology [7]. Calcification can also arise from fibroadenoma in the interstitial tissues and from arteriosclerotic changes in the arterial walls. A recent study indicated that mammography can be used to detect arterial wall calcification, which may have potential implications in the risk assessment of cardiovascular events [8]. Calcification detected by mammography can arise from various causes; therefore, interpreting the cause of calcification becomes a crucial factor for diagnosis.

When monitoring cases using mammography, the primary focus is often on changes in the size and shape of the tumor and the appearance and rate of calcification. However, this report emphasizes the clinical significance of observing reductions in arterial calcification within and around tumors. Changes in arterial calcifications may serve as a crucial indicator for the early detection of breast cancer. It is essential to be aware of this possibility in clinical practice and further investigate this phenomenon in future research.

## Figures and Tables

**Figure 1:**
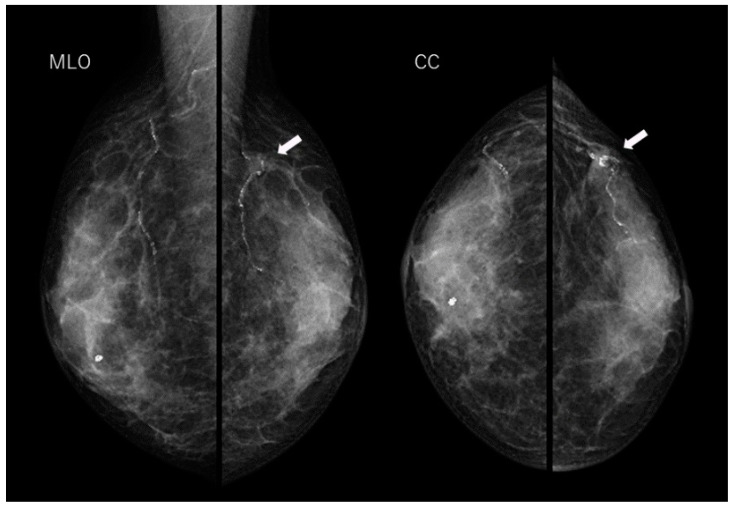
In the mediolateral oblique (MLO) and cranial caudal (CC) views of the mammography, an oval-shaped tumor measuring 9 mm is observed in the upper outer quadrant of the left breast (arrows). Calcified arteries are noted both within and surrounding this tumor.

**Figure 2:**
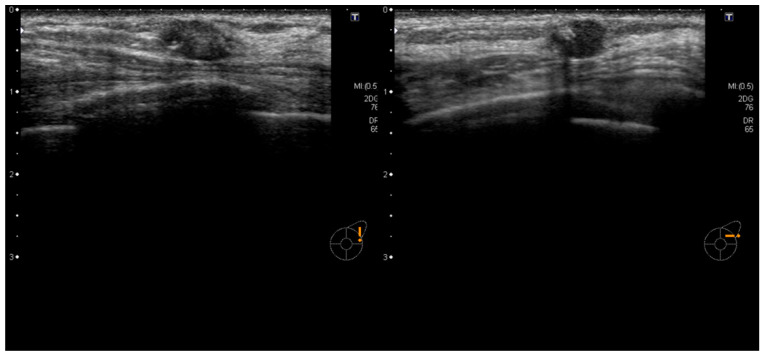
Ultrasound examination revealed a well-defined, oval-shaped tumor measuring 9 mm in the upper outer quadrant of the left breast. Fibroadenoma was suspected, and a plan was made for follow-up observation using ultrasound and mammography.

**Figure 3:**
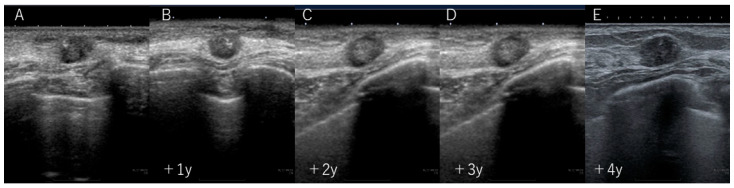
From (**A**) the initial ultrasound examination to (**B**–**D**) the follow-up ultrasound examinations 3 years later, no changes in the size or shape of the tumor were observed, and the diagnosis of fibroadenoma was upheld. (**E**) In the ultrasound examination 4 years later, the tumor size slightly increased to 11 mm.

**Figure 4:**
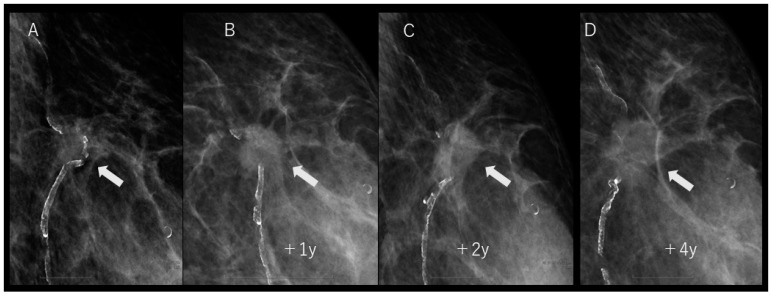
(**A**–**D**) Enlarged views of the medial oblique mammography are shown over time. (**A**) In the initial mammography, arteries with arterial calcification were observed both inside and around the tumor. However, over the years, there was a noticeable gradual disappearance of the arterial calcification (arrows).

**Figure 5:**
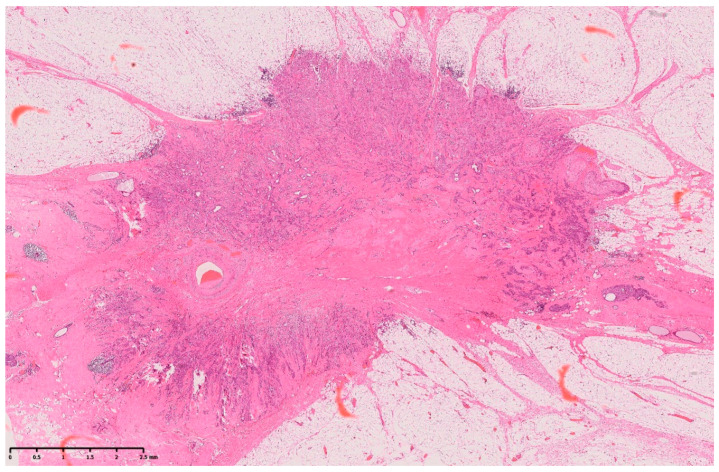
The hematoxylin- and eosin-stained specimen shows cancer cells with mildly enlarged, oval nuclei forming small cords and tubular structures, infiltrating and proliferating with fibrosis. Ultimately, a diagnosis of invasive ductal carcinoma was established (invasion diameter, 11 mm; estrogen receptor, J-Score 3b; progesterone receptor, J-Score 3b; human epidermal growth factor receptor 2, score 2; fluorescence in situ hybridization, negative; Ki-67, 7.5%).

**Figure 6:**
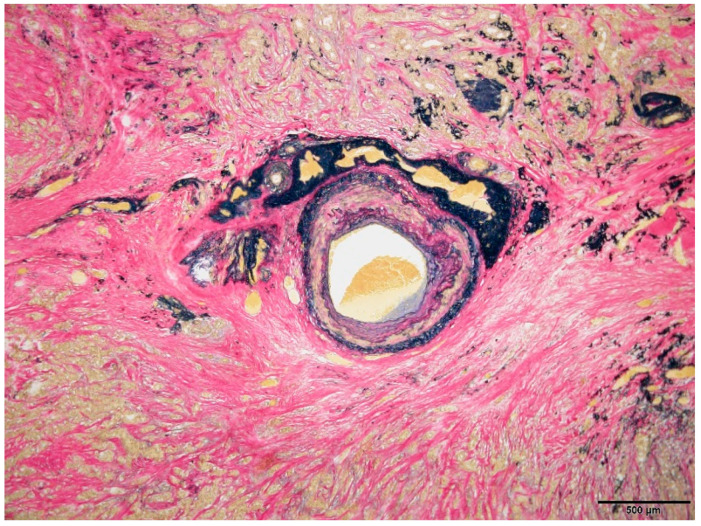
A thick artery is present in the tumor, initially thought to correspond to the calcified artery observed on mammography. Elastica van Gieson staining of this area shows that the arterial wall structure is preserved and vascular invasion by the tumor is not detected. Although calcification due to arteriosclerosis is observed in the arterial walls of the patient’s normal breast tissue, such calcifications are not found in the arteries within or surrounding the tumor.

## Data Availability

Dataset available on request from the authors.
